# Prediction and evaluation of high-risk patients with primary biliary cholangitis receiving ursodeoxycholic acid therapy: an early criterion

**DOI:** 10.1007/s12072-022-10431-7

**Published:** 2022-10-30

**Authors:** Chunmei Yang, Guanya Guo, Bo Li, Linhua Zheng, Ruiqing Sun, Xiufang Wang, Juan Deng, Gui Jia, Xia Zhou, Lina Cui, Changcun Guo, Xinmin Zhou, Patrick S. C. Leung, M. Eric Gershwin, Yulong Shang, Ying Han

**Affiliations:** 1 National Clinical Research Center for Digestive Diseases and Xijing Hospital of Digestive Diseases, Xijing Hospital, Air Force Military Medical University, Xi’anXi’an, 710032 China; 2 Division of Rheumatology, Allergy and Clinical Immunology, University of California at Davis, Davis, CA 95616 USA

**Keywords:** Autoimmune liver disease, Primary biliary cholangitis, Adverse outcome, Stratified therapy, Early prediction, Therapeutics, Prognosis, Biochemical response, Complication, Retrospective cohort study

## Abstract

**Background and aims:**

Current treatment guidelines recommend ursodeoxycholic acid (UDCA) as the first-line treatment for new-diagnosed primary biliary cholangitis (PBC) patients. However, up to 40% patients are insensitive to UDCA monotherapy, and evaluation of UDCA response at 12 months may result in long period of ineffective treatment. We aimed to develop a new criterion to reliably identify non-response patients much earlier.

**Methods:**

Five hundred sixty-nine patients with an average of 59 months (Median: 53; IQR:32–79) follow-up periods were randomly divided into either the training (70%) or the validation cohort (30%). The efficiency of different combinations of total bilirubin (TBIL), alkaline phosphatase (ALP), and aspartate aminotransferase (AST) threshold values to predict outcomes was assessed at 1, 3 or 6 month after the initiation of UDCA therapy. The endpoints were defined as adverse outcomes, including liver-related death, liver transplantation and complications of cirrhosis. Adverse outcome-free survival was compared using various published criteria and a proposed new criterion.

**Results:**

A new criterion of evaluating UDCA responses at 1 month was established as: ALP ≤ 2.5 × upper limit of normal (ULN) and AST ≤ 2 × ULN, and TBIL ≤ 1 × ULN (Xi’an criterion). The 5 year adverse outcome-free survival rate of UDCA responders, defined by Xi’an criterion, was 97%, which was significantly higher than that of those non-responders (64%). An accurate distinguishing high-risk patients’ capacity of Xi’an criterion was confirmed in both early and late-stage PBC.

**Conclusions:**

Xi’an criterion has a similar or even higher ability to distinguish high-risk PBC patients than other published criteria. Xi’an criterion can facilitate early identification of patients requiring new therapeutic approaches.

**Supplementary Information:**

The online version contains supplementary material available at 10.1007/s12072-022-10431-7.

## Introduction

Primary biliary cholangitis (PBC) is an immune-mediated liver disease characterized by chronic inflammation of the intrahepatic bile ducts that causes progressive ductal damage and liver fibrosis [1]. PBC has heterogeneous clinical features, and some patients can develop cirrhosis, hepatic failure, and liver-related death during disease progression [2, 3]. Currently, ursodeoxycholic acid (UDCA) is the first-line therapy for PBC, which can improve liver biochemistry indicators, ameliorate disease-associated symptoms and suppress liver fibrosis progression [4, 5]. However, a significant proportion of patients have an inadequate response to UDCA, which leads to a higher risk of liver-related progression [6]. To assure adequate clinical management and personal care, it is necessary to define and establish reliable parameters in identifying subgroups of patients at high risk.

In the past few decades, standard serum liver biochemistry testing under UDCA treatment has been used to predict treatment responses, and liver-related complications. Several criteria for UDCA treatment have been developed to evaluate patient risk stratification, such as Rotterdam, Barcelona, Rochester-II, Paris-I, Paris-II, Toronto and Ehime criteria [7–13]. Those prognosis risk stratification model assesses therapeutic effects using liver biochemical parameters after UDCA treatment initiation for 6, 12, or 24 months, respectively. A 12 month period is conventionally used to identify patients in needs for second-line therapies [6]. However, these criteria also posed potential limitations for patients with inadequate responses who were at a higher risk of disease progression to receive non-effective treatment for a long period.

Notably, approximately 50% of patients might need additional treatments to reach therapeutic goals [14]. The rate of progression varies greatly among individual patients [15]. Although more patients are being recognized with earlier-stage disease, there are still a considerable proportion of patients who are progressing rapidly [4, 16]. Mean survival in patients with bilirubin level of 2 mg/dL is 4 years, and that in patients with bilirubin level of 6.0 mg/dL is only 2 years [17].

In this study, we retrospectively reviewed the clinical parameters and ascertained liver-related events. To identify patients who can likely benefit from early initiation of second-line therapy, we selected biochemical indicators at different time-points and constructed a new risk stratification criterion to predict insufficient responses to UDCA treatment.

## Methods

### Study design

We collected and analyzed data from 569 patients diagnosed with PBC between 2004 and 2021 in the Xijing Hospital of the Fourth Military Medical University (Xi’an, China). The diagnosis and treatment of PBC were based on international guidelines [6, 15]. Briefly, PBC was diagnosed when at least two of the following three criteria were met: (i) biochemical evidence of cholestasis with elevation of ALP, (ii) positivity for anti-mitochondrial antibodies, and (iii) consistency with PBC in liver biopsy. All participants were treated regularly with UDCA at 13–15 mg/kg/day. We only included PBC patients who were treated with UDCA continuously for at least 1 year after the diagnosis. Patients were excluded if they had an end-point within 6 months, viral hepatitis (hepatitis B or C), alcoholic liver disease, primary sclerosing cholangitis, steatohepatitis, and overlapping autoimmune hepatitis.

### Baseline and laboratory data

Data on gender, age at diagnosis, blood tests (including alanine transaminase [ALT], AST, ALP, total bilirubin [TBIL], albumin [ALB], and platelets [PLT], immunoglobulin [Ig] G, IgM) at baseline and after 1, 3, 6, and 12 months of UDCA therapy; cirrhosis was defined based on histology or imaging evidence of cirrhosis via ultrasound, computed tomography or MRI; and liver histology stage as early (I/II) or late (III/IV) according to the Ludwig classification [18] on all subjects were obtained from Medical Records, Xijing Hospital of the Fourth Military Medical University (Xi'an, China). The data were used to calculate the UDCA response criteria and survival analysis.

### Definitions of biochemical response and endpoints

The biochemical response to UDCA treatment was evaluated according to six previously published definitions: (1) Barcelona criteria, a decrease in ALP level 40% of baseline values or a return to normal levels after 1 year of treatment; (2) Paris-I criteria, biochemical response was defined as ALP < 3 × ULN, AST < 2 × ULN, and bilirubin ≤ 1 mg/dL after 1 year of UDCA treatment; (3) Paris-II criteria, AST and ALP ≤ 1.5 × ULN, with a normal bilirubin level after 1 year of UDCA therapy; (4) Rochester-II criteria, ALP < 2 × ULN at 12 months of UDCA therapy; (5) Rotterdam criteria, normalization of abnormal albumin and/or bilirubin levels after 1 year of UDCA treatment; (6) Ehime criteria, a 70% decrease from baseline level or a normal level of GGT after 6 months of UDCA treatment.

For the present study, all the definitions mentioned above were applied and evaluated using the same endpoint, that is, the occurrence of adverse outcome as defined by at least one of the following events: liver-related death, liver transplantation, and complications of cirrhosis (namely ascites, variceal bleeding, or hepatic encephalopathy). Data were censored at the time of death or liver transplantation for the patient who died or underwent transplantation, and at the time of presenting with a cirrhosis-related complication or the last follow-up for the living non-transplanted patients. If a living non-transplanted patient developed more than one cirrhosis-related complication during follow-up, data were censored at the time of the first presentation of cirrhosis-related complications. To improve the prognostic performance of the criteria as early as possible, different cut-off values of ALP and AST levels with a normal TBIL at 1, 3, or 6 months were assessed to define new criteria.

### Statistical analysis

Quantitative variables were presented as median with interquartile range (IQR). Comparisons of the biochemical liver tests at baseline, 1-, 3-, 6-, or 12 months were performed using the Wilcoxon signed-rank test for paired data. Categorical variables were presented as counts with percentages and compared by Chi-squared test or Fisher’s exact test. Adverse outcome-free survival was estimated using the Kaplan–Meier method and compared by log-rank test. The effect of baseline variables or 1-, 3-, 6-, or 12 month biochemical response to UDCA on survival was estimated using the Cox proportional-hazards regression model. The average hazard ratio (HR) and 95% confidence interval (CI) were used to quantify the strength of the statistical links between the tested variables and survival. Univariate Cox regression analyses were applied to the training cohort to identify prognostic factors with different cut-off values of liver tests.

The C-index, likelihood ratio Chi-square, area under time-dependent receiving operator characteristic (timeROC) curve, sensitivity, specificity, positive (PPV), and negative (NPV) predictive values, as well as positive (PLR) and negative (NLR) likelihood ratios, were calculated for all definitions to assess their performance in predicting long-term outcomes. Akaike information criterion (AIC) was also calculated to compare the loss of information for different models. Bootstrapping with 1,000 samples was used for model validation. C-index and 95% CI was calculated by survcomp package by in R software. Statistical analyses were carried out using SPSS software (version 22.0; SPSS Inc., Chicago, IL, USA). The survival curve was plotted using the R 3.5.2 software with survival, and rms packages. The timeROC curve was plotted by timeROC package. All analyses were two-sided and *p* values < 0.05 were considered statistically significant.

## Results

### Characteristics of study population

**Fig. 1:**
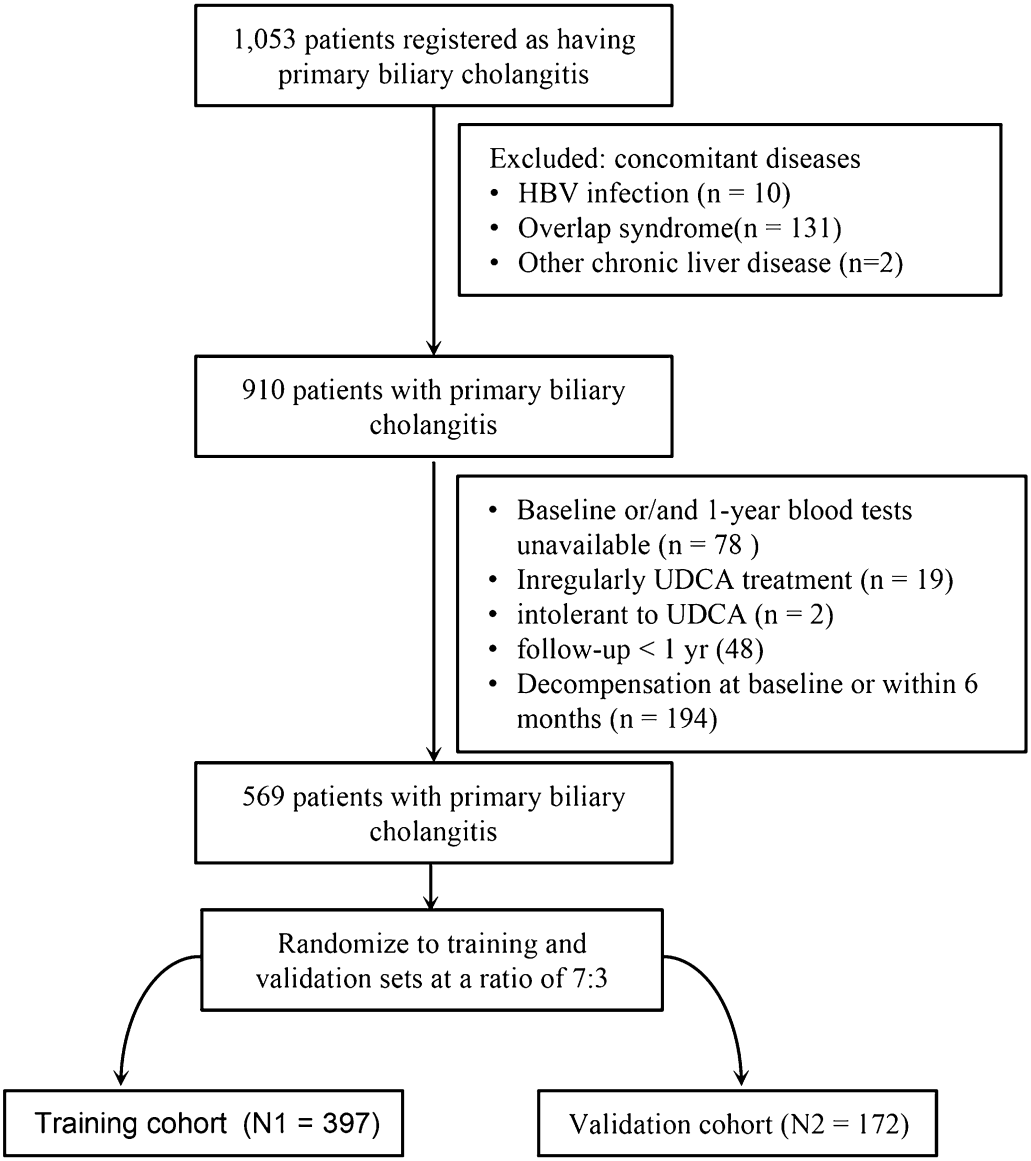
Flow chart of study design

A total of 569 patients were finally included and randomly divided into the training (*N*1 = 393) and validation (*N*2 = 176) cohort at a ratio of 7:3 (Fig. 1). Baseline characteristics were comparable between the 2 cohorts (Table 1). The median follow-up was 53 months (IQR 32–79). Among all the patients, 476 patients (84%) were female, and 387 patients were in early-stage (histological stageI–II). There were no significant differences in baseline characteristics between the training and validation cohort.

**Table 1:** Baseline demographics and clinical characteristics

Characteristics	Number (%)/Median (IQR)*			*p* value
	Entire cohort (*N* = 569)	Training cohort (*N*1 = 397)	Validation cohort (*N*2 = 172)	
Gender				0.476
Female	476 (84)	335 (84)	141 (82)	
Male	93 (16)	62 (16)	31 (18)	
Age (year)	50 (45–57)	50 (44–57)	50 (46–56)	0.988
ALP, IU/L	292 (173–480)	291 (179–485)	294 (151–475)	0.532
GGT, IU/L	325(176–549)	337 (164–568)	300 (196–511)	0.903
ALT, IU/L	65 (41–104)	69 (41–106)	62 (41–98)	0.217
AST, IU/L	67 (46–105)	66 (46–107)	68 (46–99)	0.071
ALB, g/L	39.9 (37–42.4)	39.7 (36.9–42.5)	40.1 (37.9–42.1)	0.451
TBIL, μmol/L	17.7 (12.2–30.6)	17.6 (11.9–32.0)	18.1 (12.6–28.9)	0.195
DBIL, μmol/L	7.4 (4.6–16.7)	7.3 (4.4–17.5)	7.8 (4.8–15.7)	0.125
IBIL, μmol/L	9.4 (7.1–13.0)	9.4 (6.9–12.9)	9.2 (7.4–13.1)	0.257
TBA, IU/L	15.90 (8.39–34.15)	16.51 (8.98–36.16)	14.83 (7.03–29.76)	0.172
CHO, IU/L	4.56 (3.88–5.85)	4.59 (3.90–5.87)	4.5 (3.85–5.78)	0.417
IgG, g/L	15.7 (12.8–18.7)	15.95 (13.03–18.7)	15.2 (12.2–18.8)	0.531
IgM, g/L	3.77 (2.31–5.46)	3.85 (2.35–5.47)	3.53 (2.23–5.44)	0.39
RBC, 10^12^/L	4.01 (3.71–4.32)	3.99 (3.68–4.32)	4.05 (3.77–4.32)	0.095
HGB, IU/L	122 (112–132)	122 (111–132)	122 (113–133)	0.281
PLT, 10^9^/L	151 (106–209)	155 (110–206)	147.5 (94–211)	0.951
PT, s	12.7 (12.1–13.4)	12.7 (12.1–13.4)	12.7 (12.0–13.5)	0.306
INR	0.96 (0.91–1.03)	0.96 (0.91–1.03)	0.97 (0.91–1.04)	0.847
Autoantibodies (positive, %)				
ANA	561/567 (99)	392/396 (99)	169/171 (99)	1.000
AMA	475/544 (87)	334/380 (88)	141/164 (86)	0.537
AMA-M2	370/502 (74)	250/347 (72)	120/155 (77)	0.206
gp210	187/549 (34)	131/382 (34)	56/167 (34)	0.863
sp100	64/549 (12)	50/382 (13)	14/167 (8)	0.114
Histological stage (%)^b^				0.723
Early-stage (I–II)	387(68)	271(68.3)	116(67.4)	
Late-stage (III–IV)	149(26.2)	102(25.7)	47(27.3)	
Not available	33(5.8)	24(6)	9(5.2)	
Cirrhosis (%)	158/569(28)	108/397(27)	50/172(29)	0.684
Follow-up duration (months)	53 (32–79)	52 (31–75)	55 (33–84)	0.301

*ALP* alkaline phosphatase; *ALB* albumin; *AST* aspartate aminotransferase; *ALT* alanine transaminase; *GGT* gamma-glutamyl transferase; *TBIL* total bilirubin; *DBIL* direct bilirubin; *IBIL* indirect bilirubin; *INR* international normalized ratio; *IQR* interquartile range; *PLT* platelet; *RBC* red blood cell; *IgM* immunoglobulin M; *IgG* immunoglobulin G; *TBA* total bile acids; *HGB* hemoglobin; *PT* prothrombin time; *CHO* cholesterol; *AMA* anti-mitochondrial antibody; *AMA-M2* M2 subtype of anti-mitochondrial antibody; *ACA* anti-antinuclear antibody

*Median with interquartile range are shown for quantitative variables, whereas counts with proportions are shown for categorical variables

^a^Available in 549 (94%) PBC patients and evaluated at baseline

### Adverse outcome-free survival

In entire cohort, adverse outcomes were recorded in 71 patients (12.5%), including 18 liver-related deaths, 3 liver transplantations, 50 complications of cirrhosis (30 ascites, 13 variceal bleeding, 5 with both ascites and variceal bleeding, one with hepatic encephalopathy and ascites, and one with hepatic encephalopathy, ascites, and variceal bleeding). Adverse outcome-free survival rates at 3, 5, and 10 years were 93%, 87%, and 75%, respectively (Fig. S1). Among patients with adverse outcomes, the mean time to the end-point was 3.5 years (median, 3.0 years). Importantly, 29 (41%) of them had an end-point within 2 years, and 56/71 (79%) patients within 5 years (Table 2). Among these 29 patients with an end-point with 2 years, 14 patients were in early-stage and 13 patients were in late-stage (2 patients were not available). These results showed that a considerable proportion of patients had a rapidly progress within 2 years, even in early-stage patients. Hence, considering risk stratification in these patients using the guidelines after 12 month UDCA treatment could delay their timing in receiving adjunct therapy. Therefore, we aimed to identify an earlier criterion risk stratification.

**Table 2:** Descriptive statistics of the number in 71 PBC patients with adverse outcomes over time

Time (years)	Time to adverse outcomes after diagnosis*	Number of PBC patients with adverse outcome (%)	Number according histological stages	
			Early/late-stage patients	Not available
≤ 2 years	15 (11–22)	29 (41%)	14/13	2
2 ~ 5 years	44 (37–50)	27 (38%)	9 /12	6
> 5 years	79 (72–111)	15 (21%)	4/10	1

*Median with interquartile range (IQR) were shown

### Cut-off values of biochemical parameters for risk stratification

**Fig. 2:**
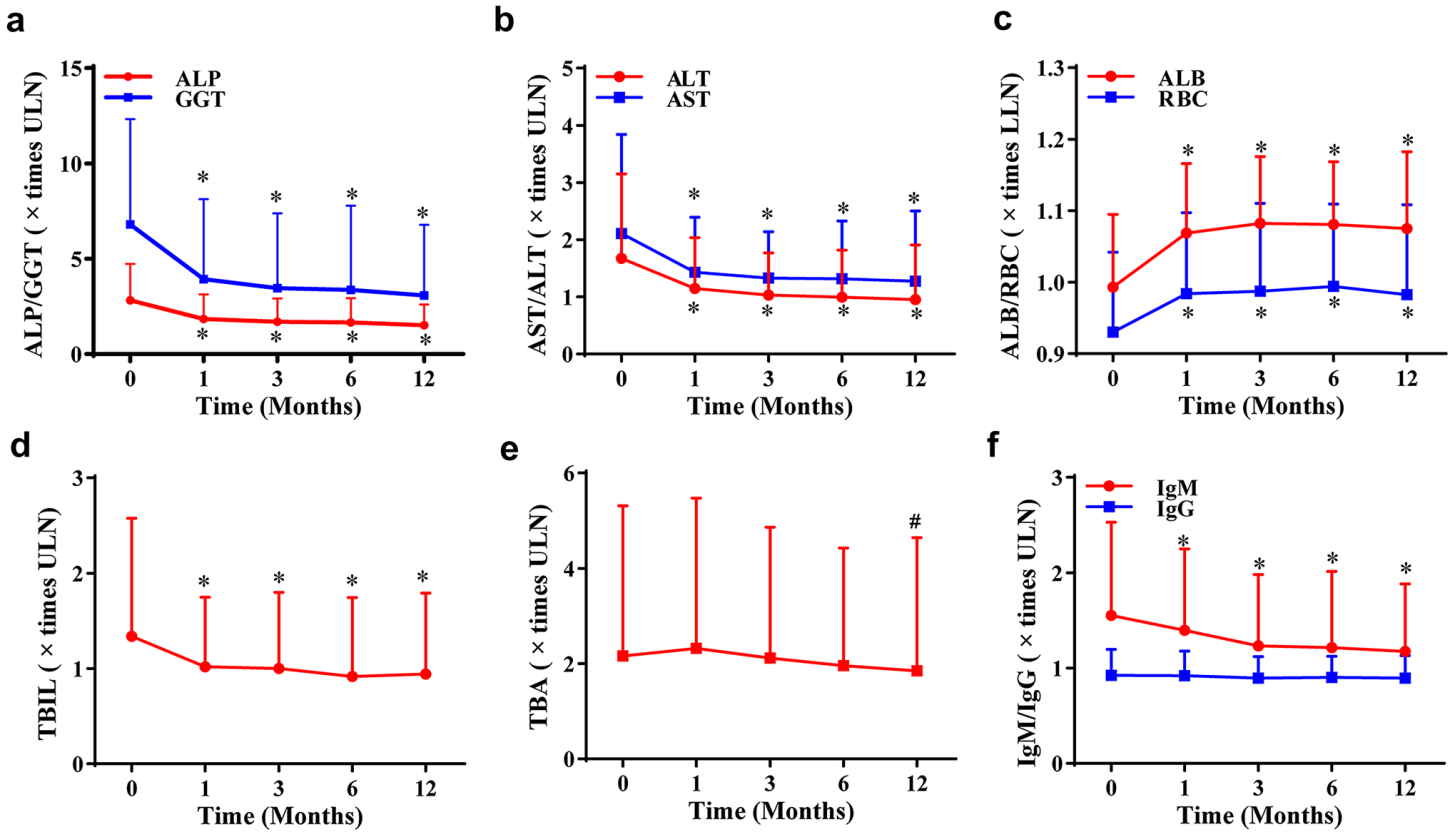
The dynamic change of the levels of ALP, GGT (**a**), AST, ALT (**b**), ALB, RBC (**c**), TBIL (**d**), TBA (**e**) and IgM/G (**f**) within 1 year in PBC patients. Data are expressed as mean ± SD. *p < .001 versus baseline. ^#^*p* < 0.05 versus baseline

We firstly analyzed the dynamic changes of biochemical indicators within 1 year in entire cohort (Fig. 2). The serum levels of ALP, GGT, AST, and ALT at 1 month decreased by ~ 40%, and TBIL decreased by ~ 25% when compared with baseline values. These biochemical values fluctuated slightly and almost remained at stable levels thereafter. In univariate cox regression analysis, biochemical parameters associated with prognosis were a serum activity of ALP ≤ 2.5 × ULN, ALP ≤  × 2ULN, AST ≤ 1.5 × ULN, AST ≤ 2.5 × ULN, and TBIL ≤ 1ULN at 1, 3, or 6 months (Table S1). Thus, we subsequently applied these the cut-off values in further analysis.

### Establishment and validation of the new early criteria

Based on decreased cut-off values of ALP, AST and TBIL serum activities, we applied four new criteria based on biochemical values at 1 month (Xi’an_1_), 3 months (Xi’an_3_), or 6 months (Xi’an_6_) in the training cohort. These new criteria at 1, 3 or 6 months were able to significantly discriminate high-risk PBC patients, using c-indices, AIC, 2-year AUROC and 5-year AUROC values (Table 3). Among these new criteria, the top three criteria of c-indices were Xi’an_6_d_ (0.74, 95% CI 0.67–0.81), Xi’an_6_b_ (0.73, 95% CI 0.67–0.80), Xi’an_1_d_ (0.72, 95% CI 0.65–0.79) and Xi’an_3_c_ (0.72, 95% CI 0.66–0.77). And the top three criteria of 5 year AUROC values were Xi’an_6_d_ (0.75, 95% CI 0.64–0.85), Xi’an_1_d_ (0.74, 95% CI 0.66–0.82), and Xi’an_6_b_ (0.73, 95% CI 0.62–0.83). Moreover, the levels of ALP, AST, and TBIL fluctuated slightly after 1-month UDCA treatment, and we tend to choose much earlier criteria. Based on the above results and much earlier judgment, we eventually selected Xi’an_1_d_ criteria (ALP ≤ 2.5 × ULN and AST ≤ 2 × ULN, and TBIL ≤ 1 × ULN), called Xi’an criterion, to discriminate prognosis.

**Table 3:** Comparison of the performance and discriminative ability of new criteria based on liver biochemical parameters at 1, 3, or 6 months in training cohort

Response definition	HR (95% CI)	p* value	2 year AUROC (95% CI)	5 year AUROC (95% CI)	LR *χ*^2^	C-index (95% CI)	AIC
Criteria based on 1-month liver biochemical parameters							
Xi’an_1_a_	5.28(2.04–13.66)	< .001	0.65(0.49–0.76)	0.69(0.59–0.76)	17	0.67(0.60–0.74)	330
Xi’an_1_b_	5.03(2.08–12.16)	< .001	0.65(0.52–0.79)	0.69(0.61–0.77)	18	0.69(0.62–0.76)	330
Xi’an_1_c_	6.67(2.58–17.25)	< .001	0.65(0.52–0.79)	0.71(0.63–0.79)	23	0.70(0.63–0.77)	325
Xi’an_1_d_	6.69(2.77–16.19)	< .001	0.68(0.55–0.81)	0.74(0.66–0.82)	25	0.72(0.65–0.79)	322
Criteria based on 3 month liver biochemical parameters							
Xi’an_3_a_	8.97(3.18–25.28)	< .001	0.71(0.62–0.81)	0.67(0.59–0.76)	29	0.71(0.65–0.76)	351
Xi’an_3_b_	4.13(1.95–8.72)	< .001	0.60(0.46–0.75)	0.64(0.55–0.74)	17	0.65(0.57–0.73)	363
Xi’an_3_c_	7.88(3.07–20.21)	< .001	0.73(0.63–0.82)	0.69(0.60–0.78)	29	0.72(0.66–0.77)	351
Xi’an_3_d_	3.68(1.82–7.42)	< .001	0.58(0.42–0.73)	0.65(0.56–0.75)	15	0.64(0.56–0.73)	365
Criteria based on 6-month liver biochemical parameters							
Xi’an_6_a_	5.95(2.44–14.53)	< .001	0.72(0.62–0.81)	0.71(0.61–0.81)	21	0.71(0.65–0.77)	295
Xi’an_6_b_	6.90(2.83–16.82)	< .001	0.74(0.65–0.84)	0.73(0.62–0.83)	25	0.73(0.67–0.80)	291
Xi’an_6_c_	4.65(2.12–10.19)	< .001	0.74(0.64–0.83)	0.72(0.62–0.83)	17	0.71(0.64–0.78)	299
Xi’an_6_d_	5.83(2.67–12.70)	< .001	0.77(0.68–0.87)	0.75(0.64–0.85)	23	0.74(0.67–0.81)	293

*AIC* Akaike information criterion; *AUROC* area under receiver operating characteristic curve; *LR* *χ*^2^ likelihood ratio chi-square; *HR* hazard ratio; *CI* confidence interval

Biochemical response was considered as a positive biochemical test without adverse outcome as an event; *p *values are based on the Cox regression analysis

*p values are based on the Cox regression analysis

^a^TBIL ≤ 1 × ULN, ALP ≤ 2 × ULN, and AST ≤ 1.5 × ULN

^b^TBIL ≤ 1 × ULN, ALP ≤ 2 × ULN, and AST ≤ 2 × ULN

^c^TBIL ≤ 1 × ULN, ALP ≤ 2.5 × ULN, and AST ≤ 1.5 × ULN

^d^TBIL ≤ 1 × ULN, ALP ≤ 2.5 × ULN, and AST ≤ 2 × ULN

In training cohort, the biochemical response rate of Xi’an criterion was 56%. The response rates of Barcelona, Paris-I, Paris-II, Rotterdam, Rochester-II, and Ehime were 67%, 68%, 44%, 66%, 79%, 45%, respectively. Notably, there were only 3.5% responders with an adverse outcome according to Xi’an criterion, which is lower than published criteria, such as Barcelona (9.6%), Paris-I (5.3%), Paris-II (4.4%), Rotterdam (5.5%), Rochester-II (8.7%), and Ehime (4.7%). The proportion of adverse outcome in non-responders of Xi’an criterion is 21.2%, which is higher than Barcelona (15.0%), Paris-II (16.9%), and Ehime (16.5%), and slightly lower than Paris-I (24.6%), Rochester-II (21.3%), Rotterdam (22.6%). Non responders judged by Xi’an criterion showed higher or at least comparable proportion of adverse outcomes compared with published criteria. But our criterion was established by the data of 1-month UDCA treatment, so we speculated that this criterion was effective.

We then further examined the Xi’an criterion using a separate cohort for validation. In validation cohort, the response rate was 54% with the Xi’an criterion. Similarly, rate of the adverse outcome in responders was only 3.9% using the Xi’an criterion when compared to 7.6–10.7% in other published criteria. Using the Xi’an criterion, the rate of adverse outcome in non-responders of Xi’an was 23.4%, which is lower than Rochester-II (27.6%). Responders defined by Xi’an criterion have a higher adverse outcome-free survival in both early- and late-stage patients in training cohort (Fig. S2A). In validation cohort, non-responders defined by Xi’an criterion had a low adverse outcome-free survival compared to responders in early-stage, while there was no statistical difference in late-stage patients (*p* = 0.063, Fig. S2B). In entire cohort, Xi’an criterion showed good discrimination both in early- and late-stage patients (Fig. S3), as well as cirrhotic and non-cirrhotic patients (Fig. S4).

### Assessment and comparison of the performance in predicting adverse outcomes by Xi’an and other published criteria

The performance and discrimination of the Xi’an and other published criteria (Barcelona, Paris-I, Paris-II, Rotterdam, Rochester-II, and Ehime) were compared. In training cohort (Table 4), Xi’an (HR in non-responders: 6.69; 95% CI 2.77–16.19; *p* < 0.001), Paris-I (HR: 4.83; 95% CI 2.50–9.32; p < 0.001), Paris-II (HR: 4.79; 95% CI 2.48–9.26; *p* < 0.001), Rotterdam (HR: 3.99; 95% CI 1.77–8.99; *p* < 0.001), Rochester-II (HR: 2.22; 95% CI 1.18–4.16; *p* < 0.05), and Ehime (HR: 3.5; 95% CI 1.44–8.51; *p* < 0.01) significantly discriminated the patients in terms of long-term outcome, except Barcelona (HR: 1.42; 95% CI 0.76–2.63; *p* = 0.270). In validation cohort (Table 4 and Fig. S3B), the hazard ratios (HRs) for Xi’an, Barcelona, Paris-I, Paris-II, Rotterdam, Rochester-II and Ehime were 6.86 (95% CI 1.98–23.77; *p* < 0.01), 2.26 (95% CI 0.92–5.6; *p* = 0.077), 3.4 (95% CI 1.38–8.35; *p* < 0.01), 2.98 (95% CI 1.17–7.58; *p* < 0.05), 2.3 (95% CI 0.88–5.99; *p* = 0.089), 3.39 (95% CI 1.38–8.31; *p* < 0.01), and 1.14 (95% CI 0.41–3.21; *p* = 0.800), respectively. These results showed that Xi’an criterion outperforms other criteria in identifying high-risk patients.

**Table 4:** Comparison of the performance and discriminative ability between the Xi’an and other published criteria

Response definition	HR (95% CI)	p* value	2 year AUROC (95% CI)	5 year AUROC (95% CI)	LR *χ*^2^	C-index (95% CI)	p** value	AIC
Entire cohort								
Xi’an	6.84(3.33–14.05)	< .001	0.72(0.64–0.8)	0.74(0.67–0.80)	38	0.72(0.67–0.78)		541
Barcelona	1.66(1.00–2.76)	0.053	0.48(0.39–0.57)	0.55(0.47–0.63)	4	0.53(0.47–0.60)	0.000	678
Paris-I	4.33(2.55–7.35)	< .001	0.67(0.57–0.76)	0.72(0.65–0.79)	32	0.69(0.63–0.76)	0.111	651
Paris-II	3.26(1.77–6.02)	< .001	0.60(0.51–0.69)	0.64(0.57–0.70)	17	0.63(0.57–0.69)	0.011	665
Rotterdam	4.09(2.39–7.01)	< .001	0.66(0.56–0.75)	0.71(0.64–0.78)	29	0.67(0.61–0.74)	0.044	647
Rochester-II	2.57(1.54–4.30)	< .001	0.55(0.46–0.65)	0.61(0.53–0.69)	12	0.60(0.53–0.66)	0.004	671
Ehime	2.30(1.19–4.43)	< .05	0.57(0.46–0.67)	0.60(0.52–0.68)	7	0.59(0.51–0.66)	0.003	499
Training cohort								
Xi’an	6.69(2.77–16.19)	< .001	0.68(0.55–0.81)	0.74(0.66–0.82)	25	0.72(0.65–0.79)		322
Barcelona	1.42(0.76–2.63)	0.270	0.43(0.33–0.54)	0.52(0.42–0.62)	1	0.51(0.43–0.59)	0.000	423
Paris-I	4.83(2.50–9.32)	< .001	0.67(0.54–0.80)	0.72(0.64–0.81)	25	0.70(0.62–0.78)	0.285	401
Paris-II	4.79(2.48–9.26)	< .001	0.59(0.46–0.71)	0.64(0.57–0.72)	15	0.64(0.57–0.71)	0.053	411
Rotterdam	3.99(1.77–8.99)	< .001	0.66(0.53–0.79)	0.75(0.66–0.83)	24	0.70(0.62–0.77)	0.216	401
Rochester-II	2.22(1.18–4.16)	< .05	0.49(0.39–0.60)	0.60(0.50–0.70)	6	0.58(0.5–0.65)	0.010	420
Ehime	3.5(1.44–8.51)	< .01	0.58(0.43–0.72)	0.64(0.55–0.73)	10	0.61(0.52–0.7)	0.027	305
Validation cohort								
Xi’an	6.86(1.98–23.77)	0.002	0.76(0.55–0.81)	0.74(0.66–0.82)	13	0.73(0.65–0.82)		153
Barcelona	2.26(0.92–5.6)	0.077	0.55(0.33–0.54)	0.61(0.42–0.62)	3	0.58(0.46–0.70)	0.064	178
Paris-I	3.40(1.38–8.35)	0.008	0.67(0.54–0.80)	0.71(0.64–0.81)	7	0.68(0.56–0.79)	0.108	174
Paris-II	2.98(1.17–7.58)	0.022	0.62(0.46–0.71)	0.63(0.57–0.72)	3	0.61(0.50–0.72)	0.045	178
Rotterdam	2.30(0.88–5.99)	0.089	0.65(0.53–0.79)	0.64(0.66–0.83)	5	0.63(0.51–0.75)	0.052	170
Rochester-II	3.39(1.38–8.31)	0.008	0.64(0.39–0.6)	0.64(0.50–0.70)	6	0.64(0.52–0.75)	0.090	175
Ehime	1.14(0.41–3.21)	0.800	0.55(0.43–0.72)	0.52(0.55–0.73)	0.1	0.55(0.42–0.67)	0.013	134

*AIC* Akaike information criterion; *AUROC* area under receiver operating characteristic curve; *LR χ*^2^, likelihood ratio chi-square; *HR* hazard ratio

*p values are based on the Cox regression analysis

**p values for c-index were calculated by survcomp package in R

**Fig. 3:**
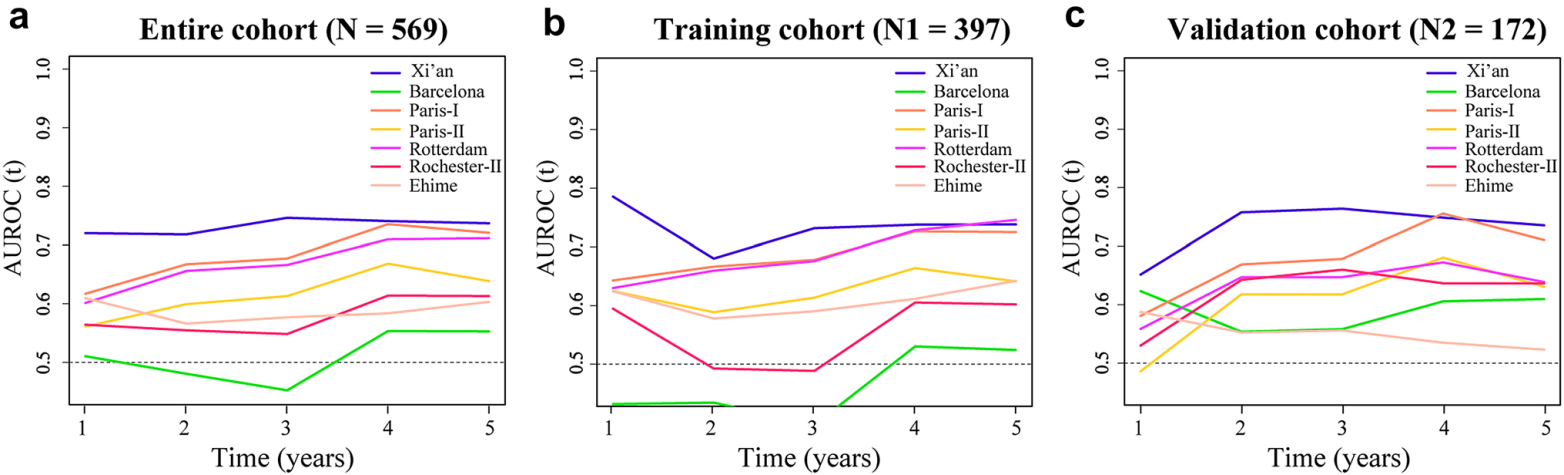
Time-dependent AUROC values of the Xi’an and other published criteria in entire cohort (**a**), training cohort (**b**) and validation cohort (**c**). *AUROC* area under receiver operating characteristic curve

The time-depended AUROC curve was shown in Fig. 3. In the entire cohort, the AUROC values of Xi’an criterion were higher than others from 1 to 5 years (Fig. 3A), as well as in training (Fig. 3B) and validation cohort (Fig. 3C), except the 5 year AUROC value of Rotterdam in training cohort, and 4 year AUROC value of Paris-I in validation cohort, with insignificant statistical difference. The HRs, likelihood ratio (LR) *χ*^2^, C-indices, AIC, 2 year AUROC, and 5 year AUROC values of Xi’an and other published criteria are shown in Table 4. In training cohort, the c-index of Xi’an (0.72, 95% CI 0.65–0.79) is higher than Barcelona (0.51, 95% CI 0.43–0.59; *p* < 0.001), Rochester-II (0.58, 95% CI 0.5–0.65; *p* < 0.05), and Ehime (0.61, 95% CI 0.52–0.70; *p* < 0.05) with significant statistical differences, and slightly higher than Paris-I (0.70, 95% CI 0.62–0.78; *p* = 0.285), Paris-II (0.64, 95% CI: 0.57–0.71; p = 0.053), Rotterdam (0.70, 95% CI 0.62–0.77; *p* = 0.216) with insignificant statistical differences (Table 4). In validation cohort, the c-index of Xi’an (0.73, 95% CI 0.65–0.82) is higher than Paris-II (0.61, 95% CI 0.5–0.72; *p* < 0.05) and Ehime (0.55, 95% CI 0.42–0.67; *p* < 0.05) with significant statistical differences, and slightly higher than Paris-I (0.68, 95% CI 0.56–0.79; *p* = 0.108), Rotterdam (0.63, 95% CI 0.51–0.75; *p* = 0.052) Barcelona (0.58, 95% CI 0.46–0.70; *p* = 0.064), and Rochester-II (0.64, 95% CI 0.52–0.75; *p* = 0.090) with insignificant statistical differences (Table 4). These results showed that, in comparison with the published criteria, the Xi’an criterion had a similar or even stronger discriminative ability to high-risk PBC patients.

A positive event was defined by a positive biochemical test without adverse outcomes. Xi’an criterion had a higher specificity (0.82) than Barcelona (0.44), Paris-I (0.68), Rochester-II (0.39), Rotterdam (0.68), and Ehime (0.81), and only lower than Paris-II (0.83) in training cohort, and had the highest specificity (0.83) than other published criteria in validation cohort (Table S2). Meanwhile, both in training and validation cohort, the PPV and PLR of Xi’an criterion were higher than other published criteria (Table S2). These results illustrated that Xi’an criterion evaluated at 1 month could be used to identify patients at high-risk accurately.

### Discrimination of high-risk patients with rapid progression by Xi’an and other published criteria

**Fig. 4:**
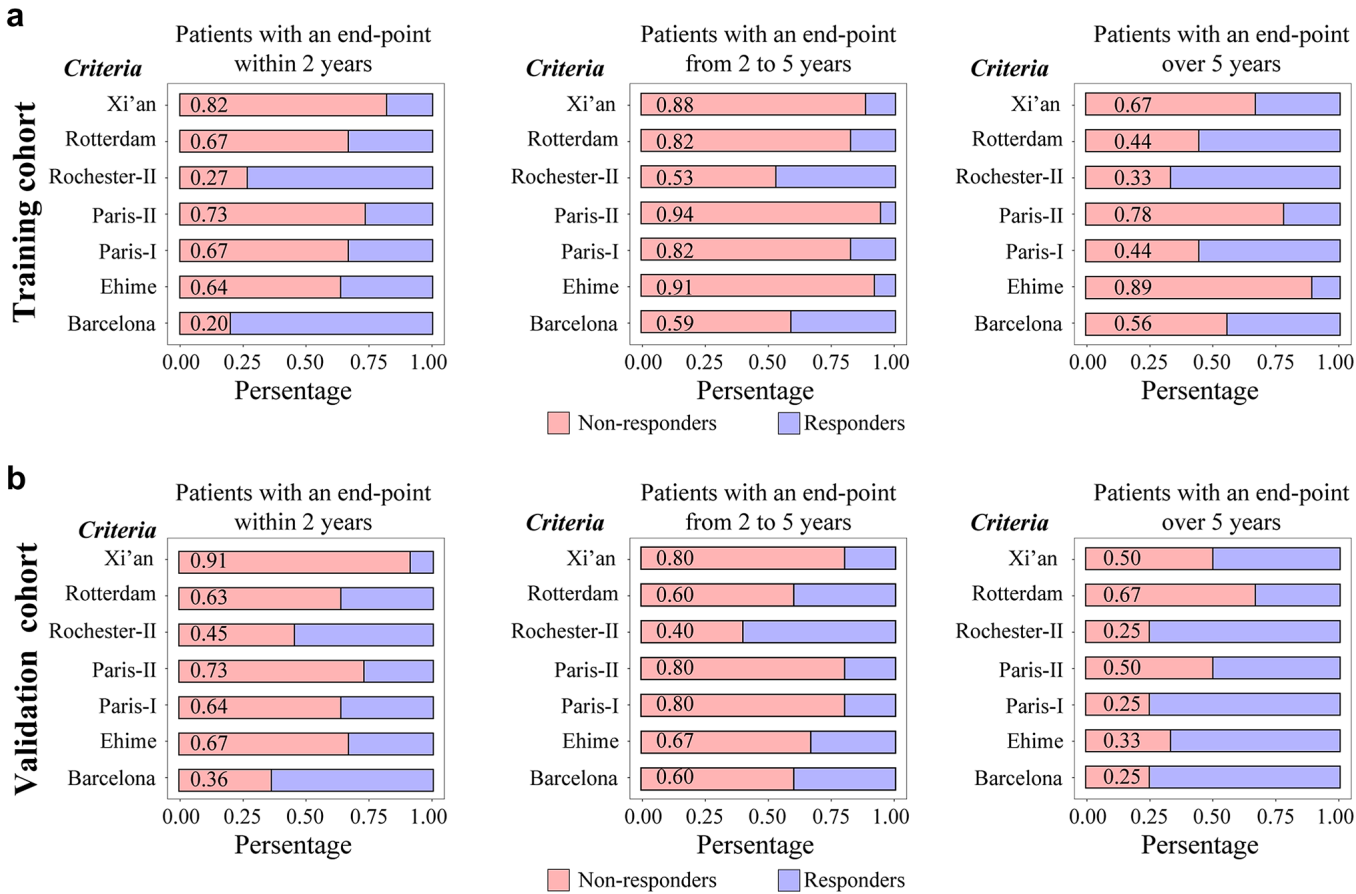
Biochemical response according different criteria in patients with adverse outcomes over time in training cohort (**a**) and validation cohort (**b**). PBC patients with an endpoints within 2 years (left), 2–5 years (median), and over 5 years after diagnosis (right)

Among the 71 patients with adverse outcome, 29/71 (41%) had an end-event within 2 years in the entire cohort. Next, we divided patients with adverse outcomes into 3 groups, including rapidly progressive (with adverse events within 2 years), moderately progressive (with adverse events from 2 to 5 years), slowly progressive patients (with adverse events over 5 years) both in training and validation cohort (Fig. 4). In training cohort, the Xi’an criterion can accurately identify 82% rapidly progressive patients, which is higher than Barcelona (20%), Paris-I (67%), Paris-II (73%), Rotterdam (67%), Rochester-II (27%), and Ehime (64%). In validation cohort, 91% rapidly progressive patients were exactly identified by Xi’an criterion, which is higher than Barcelona (36%), Paris-I (64%), Paris-II (73%), Rotterdam (63%), Rochester-II (45%), and Ehime (67%). In moderately progressive patients, Xi’an criterion could distinguish 88% patients, only lower than Paris-II (94%) and Ehime (91%) in training cohort. Furthermore, in slow progressive patients, Xi’an criterion remained effective in identifying patients with adverse events. These results showed that Xi’an criterion had a superior ability to discriminate high-risk PBC patients, especially to those who had a rapidly progression.

## Discussion

Stratified therapy is an important strategy in the clinical management of PBC patients. Several agents, such as obeticholic acid (OCA), fibrates, and budesonide proved to be effective for patients with insufficient UDCA response [19]. At present, there is also a trend to develop earlier intervention paradigms for PBC patients [3]. The clinical trial (NCT04076527) is currently ongoing to assess if OCA can improve clinical outcome in newly diagnosed PBC patients. Besides, a phase-3 clinical trial (NCT02823353) also enrolled new-diagnosed PBC patients combining fenofibrate with UDCA. In this study, we designed an earlier and excellent criterion, called Xi’an criterion, which is based on liver test using qualitative criteria after 1-month UDCA treatment, to discriminate patients who have a high risk of disease progression.

Notably, up to 40% of PBC patients will have a suboptimal biochemical response to UDCA, as assessed by binary response criteria and/or prognostic models [20]. The biochemical response to UDCA treatment strongly predicts long-term outcome. The responders defined by Paris-I criteria had a 10 year transplant-free survival rate of 90%, compared to 51% for non-responders [12]. Consequently, early identification of this subgroup patients is essential for guiding clinical practice. In this study, we determine a new definition of the biochemical response by focusing on biochemical parameters as early as possible and incorporating liver-related death, liver transplantation, and any clinical decompensated events of liver cirrhosis in the endpoints. These multiple end-point criteria are likely to better reflect the various patterns of PBC progression and be more specific to the disease course [11]. Notably, Xi’an criterion is simple qualitative criteria, like Barcelona, Paris, and Rotterdam criteria, which is much easier for clinicians to guiding clinical practice and making early prognostic judgment.

The disease progression of PBC patients varies greatly. Our data has shown the 29/71 (41%) patients with adverse outcome had an end-point within 2 years after initial diagnosis, and 82% patients in training cohort and 91% patients in validation cohort can be accurately categorized as non-responders by Xi’an criterion, which is much higher than Barcelona, Paris-I, Paris-II, Rotterdam, Rochester-II and Ehime. In a recent study by Zhang et al. [21], 47% patients with adverse outcomes had an end-point within 5 years, compared to 78% in our study. However, the proportion of late-stage patients in our study (28%; 149/527) is approximately 2 times higher than Zhang’s study (15%; 11/72). Even in early-stage, 36% (4/11) patients with adverse outcomes had an end-point within 5 years [11]. For these rapidly progressing patients, especially those who progressed within 2 years, Xi’an criterion is more effective in identifying high-risk patients than other criteria analyzed in this study. Early use of second-line agents for these high-risk patients may improve biochemical test and prolong survival without adverse outcome. Besides, Xi’an criterion had the highest c-index, specificity, PPV, and PLR both in training and validation cohort. In addition, the AUROC curve of Xi’an criterion is much higher than other published criteria. These results showed that Xi’an criterion provides an effective and reliable platform in predicting long-term outcomes.

In 2017, the EASL Clinical Practice Guidelines proposed various criteria as tools to select patients for second-line therapies and for a better design of clinical trials in PBC [6]. Multiple clinical trials were conducted to determine the safety and efficacy of other drugs such as OCA, bezafibrate, and elafibranor, in patients with incomplete response to UDCA [22–24]. Most clinical trials defined incomplete response in patients who were treated with UDCA at least for 12 months [25, 26]. Our study showed that the level of biochemical parameters used in these criteria fluctuated slightly from 1 to 12 months, and Xi’an criterion showed excellent predictive effectiveness. However, whether it is reasonable for the Xi’an criterion to define the biochemical response, apply it to the response definition of clinical research, and the guidance of PBC management and choice of second-line treatment, further research is needed.

Zhang et al. proposed that previously published criteria, including Paris, Barcelona, Toronto, and Ehime, applied at 3 and 6 months significantly discriminated high-risk patients [21]. This study shows that earlier biochemical indicators can also be used to determine the prognosis of patients. Consistent with the results of Zhang et al., biochemical parameters at 3 and 6 months in our cohort are also relevant markers in predicting poor prognosis patients. In particular, our study found that the indicators at 1 month after UDCA treatment can also effectively predict the prognosis of patients. Since Paris-I criteria is considered the best for predicting prognosis for late PBC [12], while Paris-II criteria has a better performance for early PBC [11]. We assessed the discriminatory capabilities of the Xi’an criterion at different stages, and responders defined by Xi’an criterion have a higher adverse outcome-free survival in both early- and late-stage patients in training cohort. And in validation cohort, Xi’an criterion had a good discrimination in early-stage patients, while there was no statistical difference in late-stage patients (*p* = 0.063). There were 34 late-stage patients in validation cohort, and sample size may not be large enough to pick up a statistically significant difference.

However, this study had some limitations. Firstly, it was a single-center, retrospective study. Further validation in multicenter studies with a larger cohort of patients is warranted in future. Secondly, the mean follow-up period was 5 years and relatively short. Noting the mean period of developing adverse outcome which is 3.6 years, we submit that an average of 5 years of follow-up time is sufficient to forecast the prognosis of PBC patients.

In summary, we have designed and validated a new early criterion for distinguishing high-risk PBC patients in a Chinese population for the first time. Our data indicated that PBC patients with ALP ≤ 2.5 × ULN, AST ≤ 2 × ULN, and TBIL ≤ 1 × ULN (Xi’an criterion) after 1 month UDCA treatment were likely to have better prognosis. For rapidly progressive patients, the Xi’an criterion is highly reliable and has an overall excellent predictive capacity than other published criteria. In addition, Xi’an criterion provides significant prognostic information in both early- and late-stage PBC and provides an additional comprehensive platform in the clinical evaluation of PBC patients. Most importantly, it can be readily applied in the rapid identification of PBC patients who require additional therapeutic approaches.

## Supplementary Information

Below is the link to the electronic supplementary material.

Supplementary file1 (DOCX 863 KB)

## Abbreviations

PBC: Primary biliary cholangitis
ALP: Alkaline phosphatase
AST: Aspartate aminotransferase
ALT: Alanine transaminase
GGT: Gamma-glutamyl transferase
TBIL: Total bilirubin
UDCA: Ursodeoxycholic acid
ULN: Upper limit of normal
AMA: Anti-mitochondrial antibodies
ALB: Albumin
PLT: Platelets
IgM: Immunoglobulin M
IgG: Immunoglobulin G
HR: Hazard ratio
CI: Confidence interval
AIC: Akaike information criterion
AUROC: Area under receiver operating characteristic
LR *χ*^2^: Likelihood ratio chi-square
PPV: Positive predictive values
NPV: Negative predictive values
PLR: Positive likelihood ratios
NLR: Negative likelihood ratios
SD: Standard deviation
