# Supplementary material for: Prediction and evaluation of high-risk patients with primary biliary cholangitis receiving ursodeoxycholic acid therapy: an early criterion
Source: Hepatol Int. 2022 Oct 30;17(1):237–48. doi: 10.1007/s12072-022-10431-7 (PMC9895005; doi:10.1007/s12072-022-10431-7)
Supplement: Supplementary file 1 — Supplementary file1 (DOCX 863 KB) [file 12072_2022_10431_MOESM1_ESM.docx]

| Table S1. Univariate analysis of the different cut-off values of AST, ALP and TBIL for adverse outcome-free survival in entire cohort. | | | | | | |
| --- | --- | --- | --- | --- | --- | --- |
| Variable | **At 1 month** | | **At 3 months** | | **At 6 months** | |
|  | **HR (95% CI)** | ***p* value** | **HR (95% CI)** | ***p* value** | **HR (95% CI)** | ***p* value** |
| ALP | | | | | | |
| ≤1.5×ULN | 2.82(1.55-5.15) | ＜.001 | 2.51(1.47-4.29) | ＜.001 | 2.48(1.38-4.45) | ＜.01 |
| ≤1.67×ULN | 2.74(1.55-4.86) | ＜.001 | 2.69(1.58-4.59) | ＜.001 | 2.48(1.40-4.40) | ＜.01 |
| ≤2×ULN | 2.44(1.41-4.21) | ＜.01 | 2.78(1.64-4.69) | ＜.001 | 2.48(1.4-4.37) | ＜.01 |
| ≤2.5×ULN | 2.72(1.57-4.72) | ＜.001 | 2.36(1.36-4.08) | ＜.01 | 1.97(1.02-3.79) | ＜.05 |
| ≤3×ULN | 1.99(1.09-3.63) | ＜.05 | 2.93(1.66-5.19) | ＜.001 | 1.16(0.49-2.73) | 0.73 |
| AST | | | | | | |
| ≤1.5×ULN | 2.59(1.50-4.48) | ＜.001 | 3.94(2.30-6.76) | ＜.001 | 2.69(1.53-4.72) | ＜.001 |
| ≤2×ULN | 2.16(1.21-3.87) | ＜.01 | 2.88(1.65-5.02) | ＜.001 | 2.22(1.18-4.20) | ＜.05 |
| ≤2.5×ULN | 1.42(0.61-3.34) | 0.42 | 1.91(0.90-4.05) | 0.09 | 1.58(0.67-3.74) | 0.29 |
| ≤3×ULN | 1.61(0.50-5.18) | 0.42 | 1.09(0.34-3.49) | 0.88 | 1.79(0.55-5.76) | 0.33 |
| TBIL | | | | | | |
| ≤1×ULN | 5.5(3.02-10.04) | ＜.001 | 2.95(1.74-5.01) | ＜.001 | 3.42(1.95-6.03) | ＜.001 |
| ULN, upper limit of normal; HR, hazard ratio; *p* *values* are based on the Cox regression analysis | | | | | | |

| **Table S2. Performance of biochemical response for prediction of long-term outcome.** | | | | | | |
| --- | --- | --- | --- | --- | --- | --- |
| **Response definition** | **Sensitivity** | **Specificity** | **PPV** | **NPV** | **PLR** | **NLR** |
| **Training cohort** | | | | | | |
| Xi’an | 0.61 | 0.82 | 0.96 | 0.21 | 3.48 | 0.47 |
| Barcelona | 0.68 | 0.44 | 0.90 | 0.15 | 1.21 | 0.73 |
| Paris-Ⅰ | 0.73 | 0.68 | 0.95 | 0.25 | 2.31 | 0.39 |
| Paris-Ⅱ | 0.48 | 0.83 | 0.96 | 0.17 | 2.80 | 0.63 |
| Rotterdam | 0.70 | 0.68 | 0.95 | 0.23 | 2.21 | 0.44 |
| Rochester-Ⅱ | 0.82 | 0.39 | 0.91 | 0.21 | 1.34 | 0.47 |
| Ehime | 0.48 | 0.81 | 0.95 | 0.16 | 2.57 | 0.64 |
| **Validation cohort** | | | | | | |
| Xi’an | 0.60 | 0.83 | 0.96 | 0.23 | 3.59 | 0.48 |
| Barcelona | 0.76 | 0.40 | 0.89 | 0.21 | 1.27 | 0.59 |
| Paris-Ⅰ | 0.70 | 0.60 | 0.92 | 0.23 | 1.74 | 0.51 |
| Paris-Ⅱ | 0.49 | 0.70 | 0.92 | 0.17 | 1.64 | 0.73 |
| Rotterdam | 0.64 | 0.63 | 0.92 | 0.20 | 1.75 | 0.56 |
| Rochester-Ⅱ | 0.84 | 0.40 | 0.90 | 0.28 | 1.40 | 0.40 |
| Ehime | 0.44 | 0.60 | 0.89 | 0.13 | 1.09 | 0.94 |
| *Note:* Biochemical complete response was considered as a positive biochemical test without adverse outcome as an event.  PPV, positive predictive values; NPV, negative predictive values; PLR, positive likelihood ratios; NLR, negative likelihood ratios; | | | | | | |

**
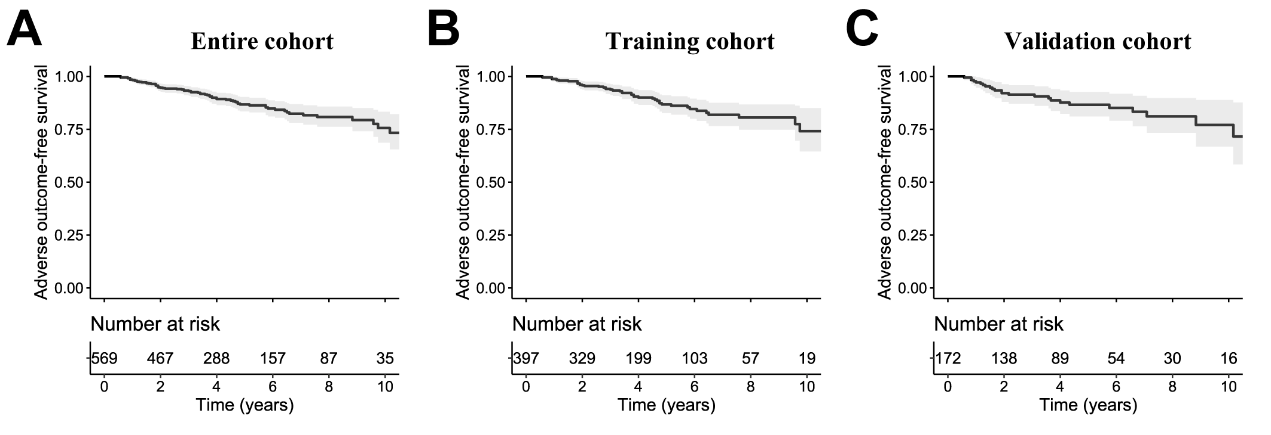
**

**Figure S1. Adverse outcome-free survival (an adverse outcome being defined by liver-related death, liver transplantation, complications of cirrhosis including ascites, variceal bleeding, and hepatic encephalopathy) in entire (A), training (B) and validation cohort (C).**

**
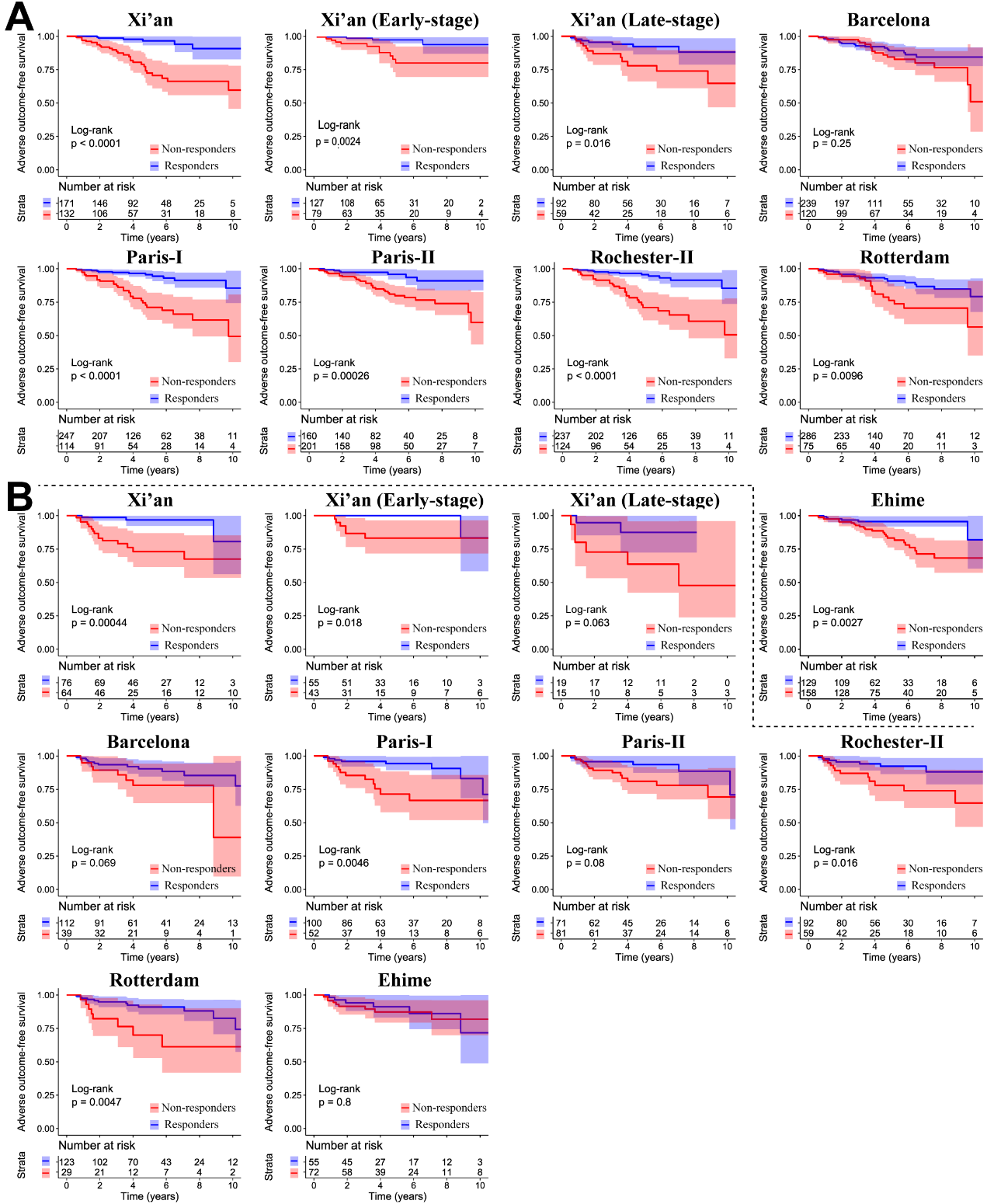
Figure S2. Adverse outcome-free survival according to biochemical response as defined by Xi’an and published criteria in training cohort (A) and validation cohort (B).** The red line represents non-responders, and the blue line represents responders.


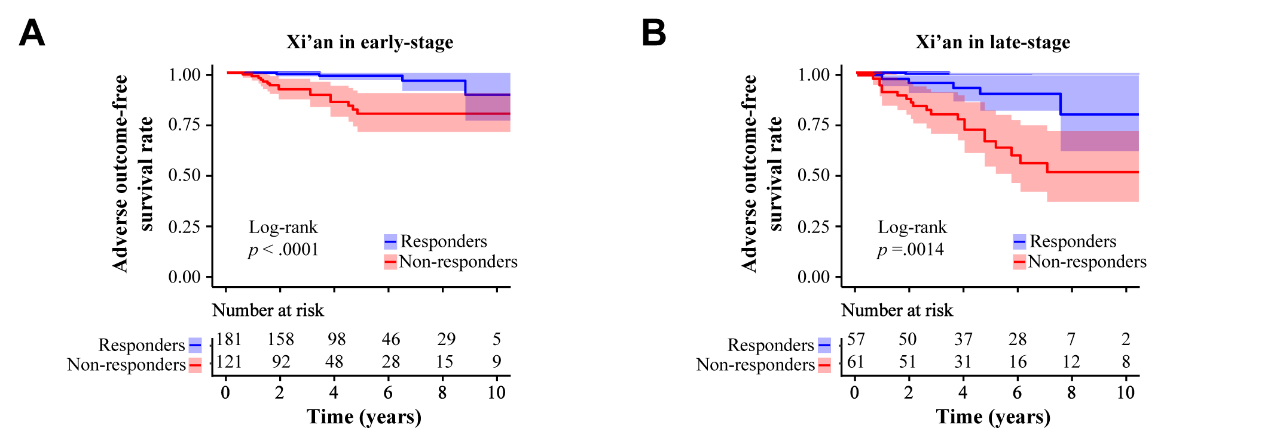


**Figure S3. Adverse outcome-free survival according to biochemical response as defined by Xi’an in early-stage (A) and late-stage patients (B) in entire cohort.** The red line represents non-responders, and the blue line represents responders.


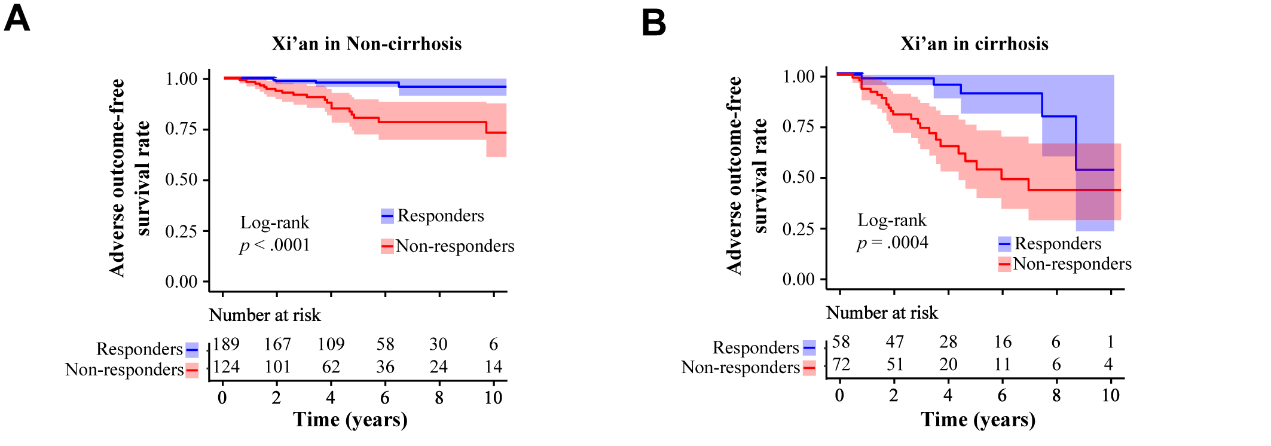


**Figure S4. Adverse outcome-free survival according to biochemical response as defined by Xi’an in non-cirrhosis (A) and cirrhosis patients (B) in entire cohort.** The red line represents non-responders, and the blue line represents responders.
